# Beta-Elemene Blocks Epithelial-Mesenchymal Transition in Human Breast Cancer Cell Line MCF-7 through Smad3-Mediated Down-Regulation of Nuclear Transcription Factors

**DOI:** 10.1371/journal.pone.0058719

**Published:** 2013-03-14

**Authors:** Xian Zhang, Yinghua Li, Yang Zhang, Jincheng Song, Qimin Wang, Luping Zheng, Dan Liu

**Affiliations:** 1 Department of Oncology, the Second Affiliated Hospital of Dalian Medical University, Dalian, China; 2 Research Institute of Integrated Traditional and Western Medicine of Dalian Medical University, Dalian, China; 3 Graduate Institute of Dalian Medical University, Dalian, China; 4 Department of Pathology, the Second Affiliated Hospital of Dalian Medical University, Dalian, China; University of Birmingham, United Kingdom

## Abstract

Epithelial-mesenchymal transition (EMT) is the first step required for breast cancer to initiate metastasis. However, the potential of drugs to block and reverse the EMT process are not well explored. In the present study, we investigated the inhibitory effect of beta-elemene (ELE), an active component of a natural plant-derived anti-neoplastic agent in an established EMT model mediated by transforming growth factor-beta1 (TGF-β1). We found that ELE (40 µg/ml ) blocked the TGF-β1-induced phenotypic transition in the human breast cancer cell line MCF-7. ELE was able to inhibit TGF-β1-mediated upregulation of mRNA and protein expression of nuclear transcription factors (SNAI1, SNAI2, TWIST and SIP1), potentially through decreasing the expression and phosphorylation of Smad3, a central protein mediating the TGF-β1 signalling pathway. These findings suggest a potential therapeutic benefit of ELE in treating basal-like breast cancer.

## Introduction

Breast cancer is the most commonly diagnosed cancer, and the second leading cause of cancer-related deaths in US women [1]. Metastasis is often a lethal component of breast cancer, while other patients die from long-term recurrence [2]. Several steps are involved in cancer metastasis, including epithelial-mesenchymal transition (EMT), invasion, intravasation, adhesion, extravasation and mesenchymal-epithelial transition (MET) [3], [4]. Recently, there has been growing interest in investigating the role of EMT in cancer metastasis, as it is the first step in the migration of breast cancer cells [5], [6], [7], [8]. Recent evidence indicates that EMT is associated with cancer cell stemness, chemoresistance and circulating tumor cells in breast cancer [9], [10], [11], [12]. Thus, inhibition of EMT may provide therapeutic potential for improving the prognosis of breast cancer patients.

EMT, essential for physiological processes, is triggered by many stimuli including tumor–stromal cell interactions, hypoxia and growth factors, including transforming growth factor-β (TGF-β) [8], [13], [14], [15]. EMT is defined by the loss of epithelial characteristics accompanied by a mesenchymal phenotype. This phenotype is characterized by reduced expression of epithelial markers (E-cadherin and β-catenin) and elevated expression of mesenchymal markers (N-cadherin, fibronectin and vimentin) [6], [16]. These proteins are mainly regulated by transcription repressors, including Snail/SNAI1, Slug/SNAI2, TWIST and SIP1, a master regulator of EMT [17], [18], [19]. TGF-β, Wnt*-*, Notch-, Hedgehog-, and NF-κB- dependent pathways can induce and maintain EMT [20], and are often considered targets to block EMT [21]. Interestingly, some natural products, including fucoidan, benzyl isothiocyanate, curcumin, 2-hydroxycinnamaldehyde can block EMT by regulating miRNA [22], [23], [24], [25].

Elemene (1-methyl-1-vinyl-2, 4-diisopropenyl-cyclohexane), an active component of the herbal medicine Curcuma wenyujin, is a novel anti-cancer drug [26]. An extract of elemene contains a mixture of α-, β- and δ-elemene, in which the main component β-elemene (ELE) accounts for 60∼72% of total drug [27]. ELE has been clinically used to treat leukaemia and carcinomas in brain, breast, liver and other tissues [26], [28], [29], [30], as one of its formulations has been approved by the State Food and Drug Administration of China for treating primary and secondary brain tumors. In addition, ELE induces apoptosis as well as protective autophagy in human non-small-cell lung cancer A549 cells [31], induces apoptosis in bladder cancer T24 cells via downregulation of survivin, Bcl-xL and Mta-1 expression [32], and regulates the activities of Ras/Raf/MEK/ERK and the AKT cascades [33], [34]. Collectively, these studies indicate that ELE has apoptosis-inducing activity and regulates signalling pathways critical for cell survival in *in vitro* systems. However, it is unclear whether ELE plays a role in EMT in human breast cancer cells.

Using an EMT model of the breast cancer cell line MCF-7 treated with TGF-β1, we examined the effect of ELE on EMT-related phenotypic and gene expression changes.

## Materials and Methods

### Chemicals and Reagents

ELE (98% purity) was purchased from Dalian Jingang Pharmaceuticals, Ltd (Liaoning, China). The main component in the ELE is β-elemene, with a molecular formula of C_15_H_24_ and molecular weight of 204.35. There are only small fractions of γ-elemene and δ-elemene, whose molecular formulas are C_16_H_26_/C_15_H_24_ and molecular weights are 218.39/204.35, respectively. The biologically available concentration of β-elemene is unclear.

Primary antibodies against E-cadherin, N-cadherin, vimentin, β-catenin, SNAI1/2, TWIST, SIP1, Smad3, p-Smad3 and β-actin were purchased from Abcam Biotechnology (Cambridge, UK). The secondary horseradish peroxidase (HRP)-conjugated goat anti-rabbit-IgG and HRP-conjugated goat anti-mouse-IgG antibodies were purchased from Santa Cruz Biotechnology (Santa Cruz, CA, USA).

### Cell Line and Drug Treatment

The human breast cancer cell line MCF-7 was obtained from the Cell Bank of the Chinese Academy of Sciences (Shanghai, China) and cultured in DMEM/high glucose, 20% fetal bovine serum (FBS), with 1% penicillin/streptomycin (Gibco, Carlsbad, CA, USA). MCF-7 was cultured at 37°C in a humidified incubator (Heraeus, Germany) supplemented with 5% CO_2_ and seeded at 2.5×10^5^ cells/ml in 6-well plates (Corning, NY, USA). Cells were maintained in serum-free DMEM/high glucose for 24 hrs, followed by treatment with 10 ng/ml TGF-β1 (R&D Systems, Minneapolis, MN, USA) [35]. Cells were examined in four groups: treated with TGF-β1 and ELE (+TGF-β1/+ELE); TGF-β1 alone (+TGF-β1/−ELE); ELE alone (−TGF-β1/+ELE); or no treatment (−TGF-β1/−ELE).

### Western Immunoblot Analysis

MCF-7 cells were washed with ice-cold PBS in duplicate and solubilised in 1% Triton lysis buffer [1% Triton X-100, 50 mmol/L Tris-Cl (pH 7.4), 150 mmol/L NaCl, 10 mmol/L EDTA, 100 mmol/L NaF, 1 mmol/L Na_3_VO_4_ (Sigma-Aldrich, Saint Louis, MO, USA), 1 mmol/L PMSF and 2 µg/mL aprotinin] on ice, followed by quantification using the Lowry method. Cell lysates (50 µg) were separated using sodium dodecyl sulfate-polyacrylamide gel electrophoresis and transferred to PVDF membranes (Immoblin-P; EMD Millipore, Billerica, MA, USA). The membranes were blocked with 5% bovine serum albumin (Sigma-Aldrich, Saint Louis, MO, USA) at RT for 1 hr and incubated overnight at 4°C with the primary antibodies: E-cadherin (1∶1000), β-catenin (1∶5000), N-cadherin (1∶1000), vimentin (1∶5000), SNAI1 (1∶500), SNAI2 (1∶1000), TWIST (1∶50), SIP1 (1∶100), Smad3 (1∶1000), p-Smad3 (1∶1000) and β-actin (1∶200). The membranes were incubated with the appropriate HRP-conjugated secondary antibodies for 30 mins at RT following incubation with Tris Buffered Saline with Tween-20 (TBST) buffer. The signals were visualized with enhanced chemiluminescence reagent (Beyotime Biotechnology, Nantong, China). Images were analysed using the NIH Image J software.

### Real-time PCR (RT-PCR)

Total RNA was extracted through homogenization in 1 mL TRIzol reagent (Invitrogen, Carlsbad, CA, USA), followed by chloroform extraction and isopropanol precipitation. Real-time PCR was performed in a 20 µL mixture containing 1.6 µL of cDNA template, 1 µL of each primer (10 mM), and 10 µL of a SYBR Green master mix (Takara, Dalian, China). Primers used included: 5′-TCAGACGAGGACAGTGGGAAAG-3′ (sense) and 5′- GCTTGTGGAGCAGGGACATTC-3′ (antisense) for SNAI1; 5′-CTTCCTGGTCAAGAAGCA-3′ (sense) and 5′-GGGAAATAATCACTGTATGTGTG-3′ (antisense) for SNAI2; 5′-TCTCGGTCTGGAGGATGGAG-3′ (sense) and 5′-GTTATCCAGCTCCAGAGTCT-3′ (antisense) for TWIST; 5′-AGCCGATCATGGCGGATGGC-3′ (sense) and 5′-TTCCTCCTGCTGGGATTGGCTTG-3′ (antisense) for SIP1; 5′-ACCAGGGCTTTGAGGCTGTC-3′ (sense) and 5′-GCAAAGGCCCATTCAGGTG-3′ (antisense) for Smad3; 5′- GCACCGTCAAGGCTGAGAAC-3′ (sense) and 5′- TGGTGAAGACGCCAGTGGA-3′ (antisense) for human GAPDH. The average expression levels of genes were normalized to the housekeeping gene GAPDH.

### Wound Healing Assay

MCF-7 cells were seeded in 10% FBS medium on 6-well plates at a density of 2×10^5^ cells/well. Cells were either incubated with TGF-β1 or PBS for 24 hrs as positive and negative controls, respectively. Then, cells reaching sub-confluence were scraped and allowed to migrate in serum-free medium for 12 hrs. For ELE-mediated effects, cells were incubated simultaneously with 10 ng/ml TGF-β1 and 40 µg/ml ELE for 24 hrs and subsequently scraped. The cell migration images were photographed at 0, 6, and 12 hrs following scraping. Three to four different locations were visualized and photographed under a Nikon Eclipse TE2000-S phase-contrast inverted microscope (Nikon, Japan). Three replicates of two independent experiments were averaged.

### Transwell Migration and Invasion Assay

Cell invasion was measured using a transwell chamber coated with 1–2 mg/ml Matrigel (reconstituted basement membrane; BD Biosciences, Mississauga, Canada). MCF-7 cells (2×10^5^ per transwell) were added, followed by incubation with 10 ng/ml TGF-β1, in combination with ELE (20, 40, 80 µg/ml) or PBS, at 37°C in an incubator supplemented with 5% CO_2_. Cells in the upper chamber were removed with a cotton swab after 24 hrs. The remaining cells on the membrane were fixed using methanol for 10 mins, followed by stained with 1% crystal violet solution and washed with PBS. The number of invaded cells was counted per field of view at 200× magnification.

### Cell Viability Assay

Cell viability was measured using a 3-(4, 5-dimethylthiazol-2-yl)-2, 5-diphenyltetrazolium bromide (MTT) assay. Cells were seeded at 5×10^4^ cells/well in 96-well plates, incubated overnight and then exposed to the indicated concentrations of ELE for the indicated time periods. Thereafter, 20 µl of MTT (Sigma-Aldrich, Saint Louis, MO, USA) solution (5 mg/mL) was added to each well, and cells were incubated for another 4 hrs at 37°C. Following removal of the culture medium, cells were lysed in 200 µl of dimethylsulfoxide (DMSO), and optical density (OD) was measured at 570 nm in a microplate reader (Model 550; Bio-Rad Laboratories, Hercules, CA, USA). The following formula was used: cell viability = (OD of the experimental sample/OD of the control group)x 100%.

### Statistical Analyses

The experiments were repeated at least in triplicate. Data are expressed as the mean ± SD. Differences in the results for two groups were evaluated using a Student’s t-test. P<0.05 was considered to be statistically significant.

## Results

### TGF-β1 Induces Phenotypic Transition of MCF-7 Cells

**Figure 1:**
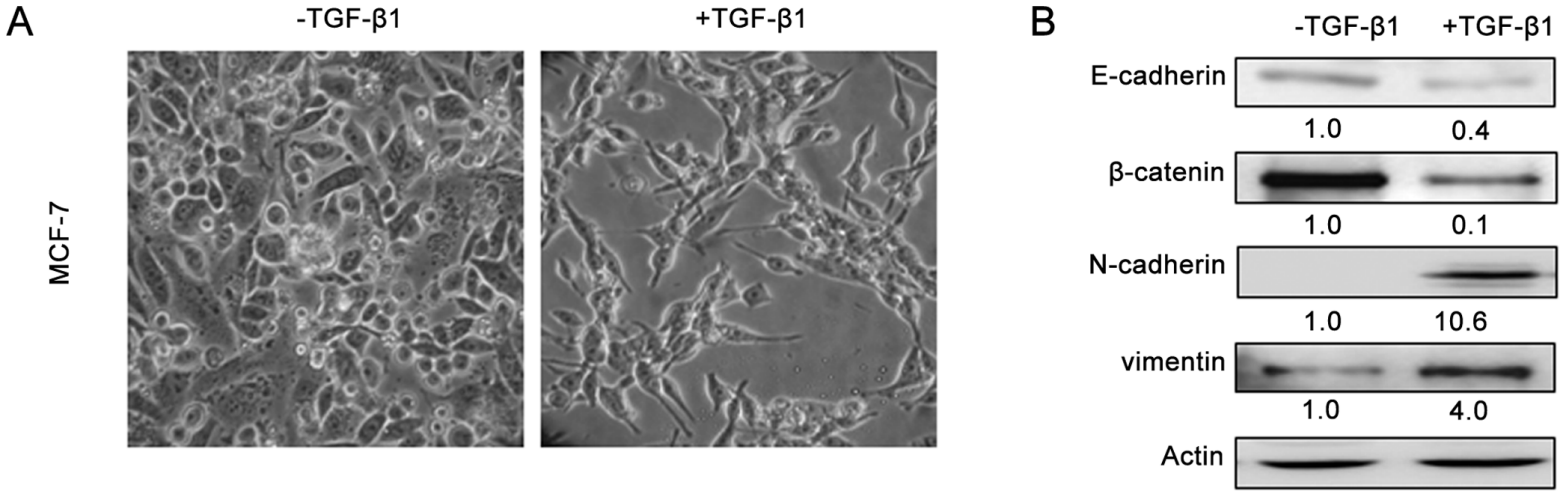
TGF-β1 induces epithelial-to-mesenchymal changes in MCF-7 cells. (A) MCF-7 cells treated with 10 ng/ml TGF-β1 for 24 hrs had a spindle-like morphology and lost intercellular junctions. Magnification, 200×. (B) Western immunoblot analysis of expression of EMT-related proteins. Expression levels of E-cadherin and β-catenin (epithelial markers) in TGF-β1-treated MCF-7 cells were markedly decreased, whereas expression levels of N-cadherin and vimentin (mesenchymal markers) were dramatically increased. TGF-β1: transforming growth factor-β1.

We initially attempted to establish an EMT model using a cobblestone-like epithelial breast cancer cell line (MCF-7 treated with 10 ng/ml TGF-β1 for 24 hrs). Consistent with previous studies, TGF-β1-treated MCF-7 cells reduced cell–cell interactions and became fibroblast-like, an EMT-like phenotypic change (Fig. 1A). Western immunoblot analysis showed a marked decrease in E-cadherin and β-catenin expression, and an increase in expression of N-cadherin and vimentin, a characteristic of EMT (Fig. 1B). These data confirmed that TGF-β1 treatment results in EMT in MCF-7 cells.

### TGF-β1 Facilitates Migration and Invasion of MCF-7 Cells

**Figure 2:**
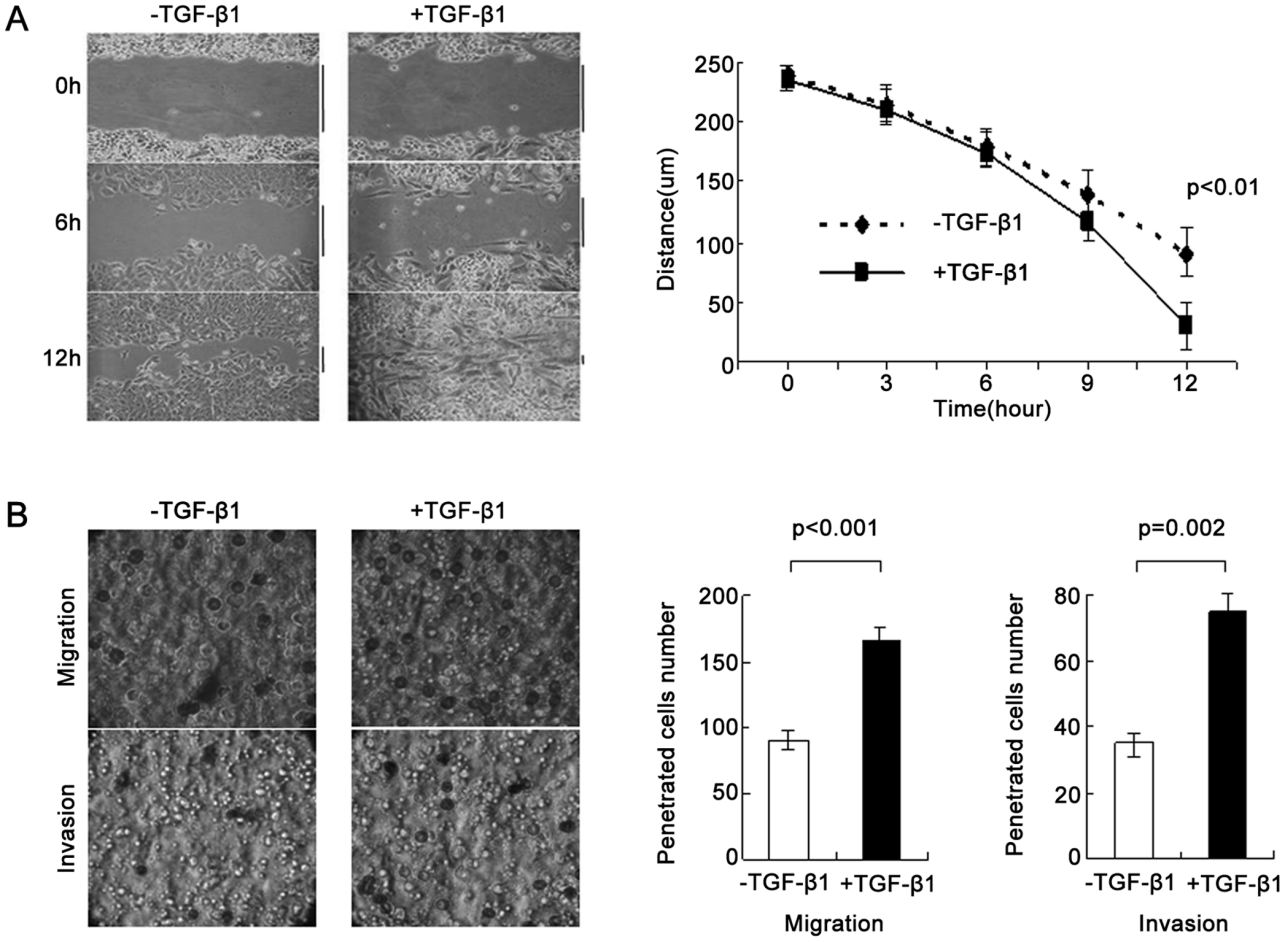
MCF-7 cells acquire migration and invasion abilities in response to TGF-β1 treatment. (A) Representative pictures of the scratch wound assay. TGF-β1-treated MCF-7 cells migrated faster than controls. The black lines indicated size of the remaining wound. Data from three independent experiments were analyzed and presented as mean ± SD. (P<0.001). (B) Representative pictures in a transwell migration assay (top).The penetrated cells numbers of MCF-7 treated with TGF-β1 for 12 hrs were significantly increased as compared to controls without TGF-β1 (P<0.001). Representative pictures in a transwell invasion assay (bottom). TGF-β1 treatment led to more MCF-7 cells penetrating matrix than controls. (P = 0.002). The results in both transwell migration and invasion assays were obtained from three independent experiments.

As mesenchymal cells move faster than epithelial cells, we next examined the migration and invasion abilities of TGF-β1-treated MCF-7 cells *in vitro*. In a wound healing assay, MCF-7 cells treated with TGF-β1 for 24 hrs migrated faster than parental MCF-7 cells (P<0.01) and closed the gap 12 hrs after generation (Fig. 2A). Using a transwell assay, TGF-β1 treatment led to more MCF-7 cells migrating and invading the membrane matrix than negative controls (P<0.001 and P = 0.002, respectively) (Fig. 2B).

### ELE Blocks TGF-β1-induced EMT in MCF-7 Cells

**Figure 3:**
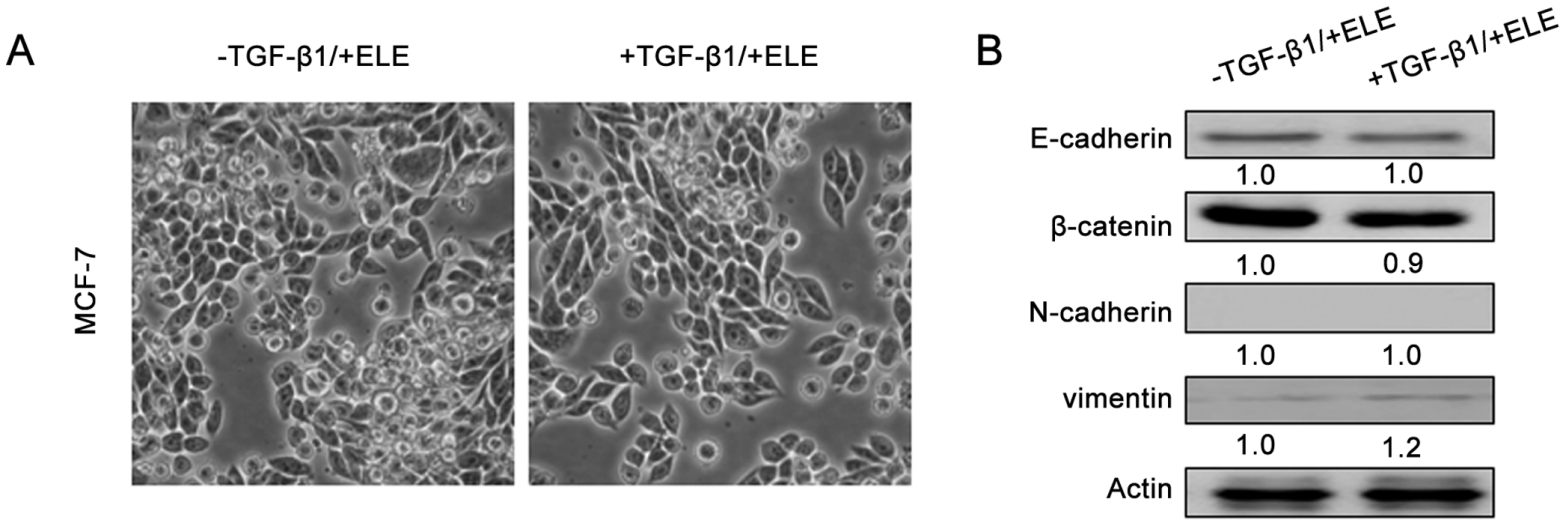
ELE blocks TGF-β1-induced EMT in MCF-7 cells. (A) Representative pictures of MCF-7 cells treated with ELE showed that epithelial morphology of cells was maintained even in the presence of TGF-β1. Magnification, 200×. (B) Western immunoblot analysis showed that expression levels of E-cadherin and β-catenin (epithelial markers) and N-cadherin and vimentin (mesenchymal markers) did not differ in cells treated with TGF-β1 and ELE compared to cells with ELE only.

We next assessed the effect of ELE on TGF-β1-mediated changes of cell morphology and gene expression in MCF-7 cells. Since the MTT assay showed that the IC50 of ELE was 534 µg/ml in TGF-β1-treated MCF-7 cells and 275 µg/ml in parental MCF-7 cells (Figure S1), MCF-7 cells were treated with 10 ng/ml TGF-β1 and 40 µg/ml ELE for 24 hrs. Surprisingly, an epithelial morphology of MCF-7 cells was maintained even in the presence of TGF-β1 (Fig. 3A). Consistent with the morphological observation, expression levels of epithelial E-cadherin and β-catenin and mesenchymal N-cadherin and vimentin were comparable between TGF-β1-treated cells and controls (Fig. 3B). These data indicate that ELE inhibits TGF-β1-induced EMT.

### ELE Inhibits TGF-β1-induced Migration and Invasion of MCF-7 Cells

**Figure 4:**
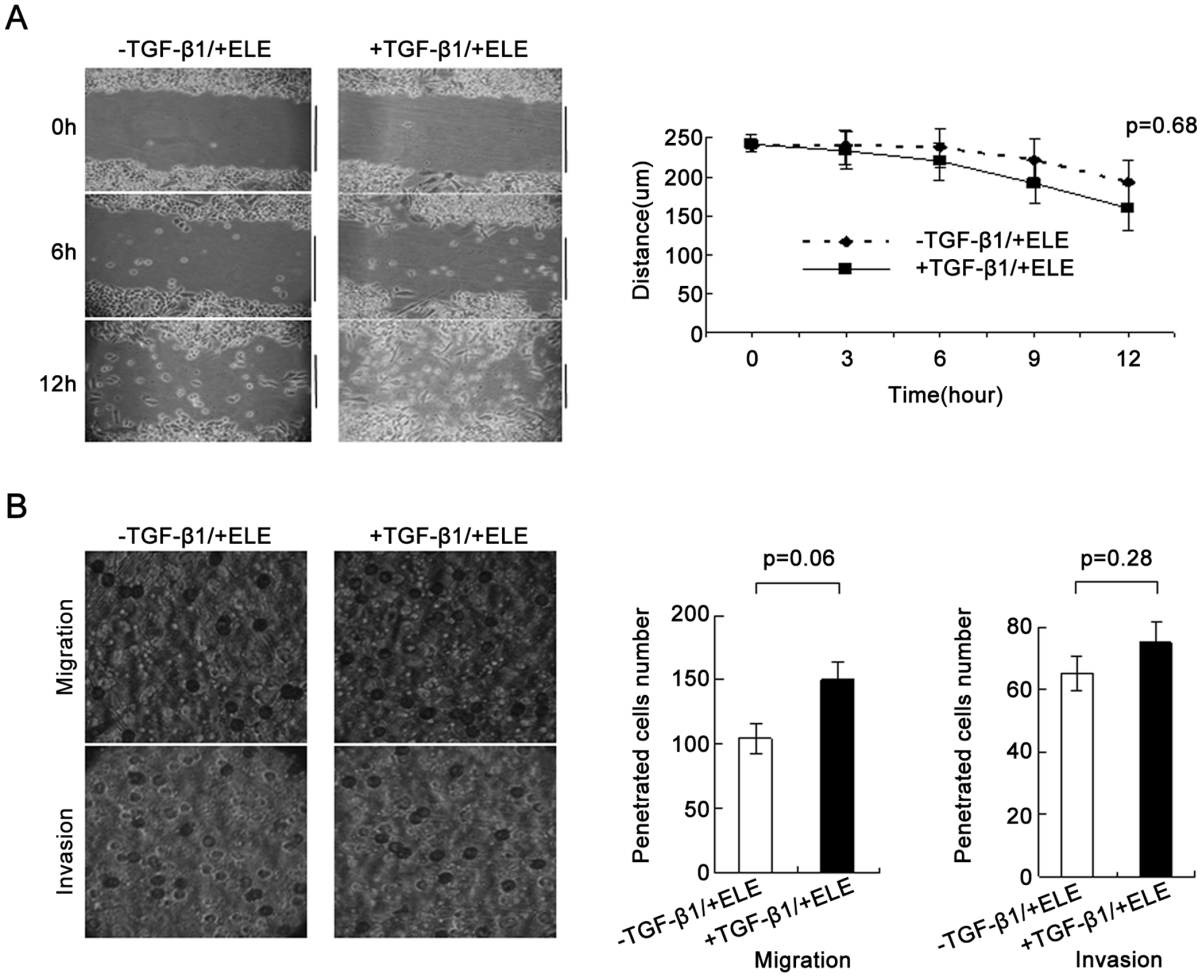
The TGF-β1-induced migration and invasion abilities of MCF-7 cells were blocked by ELE. (A) A wound healing assay showed that the migration and invasion abilities of MCF-7 cells with TGF-β1 and ELE did not differ from those of control cells without TGF-β1 (P = 0.68). The black lines showed the size of the remaining wounds. (B) Transwell migration and invasion assays demonstrated that the migration and invasion abilities of MCF-7 cells with TGF-β1 and ELE were comparable with those of cells with ELE alone (P = 0.06, and 0.28, respectively). Data are shown from three independent experiments.

MCF-7 cells treated TGF-β1 and ELE did not close the wound after 12 hrs, similar to MCF-7 cells with ELE alone (P = 0.68, Fig. 4A). The blockage effect of ELE on TGF-β1-dependent migratory ability was confirmed in the transwell assays where the numbers of MCF-7 cells treated with ELE and TGF-β1 migrating and invading the matrix were comparable to cells with ELE alone (P = 0.06 and 0.28, respectively, Fig. 4B).

### ELE Represses TGF-β1-mediated Upregulation of Transcriptional Factors that Modulate EMT

**Figure 5:**
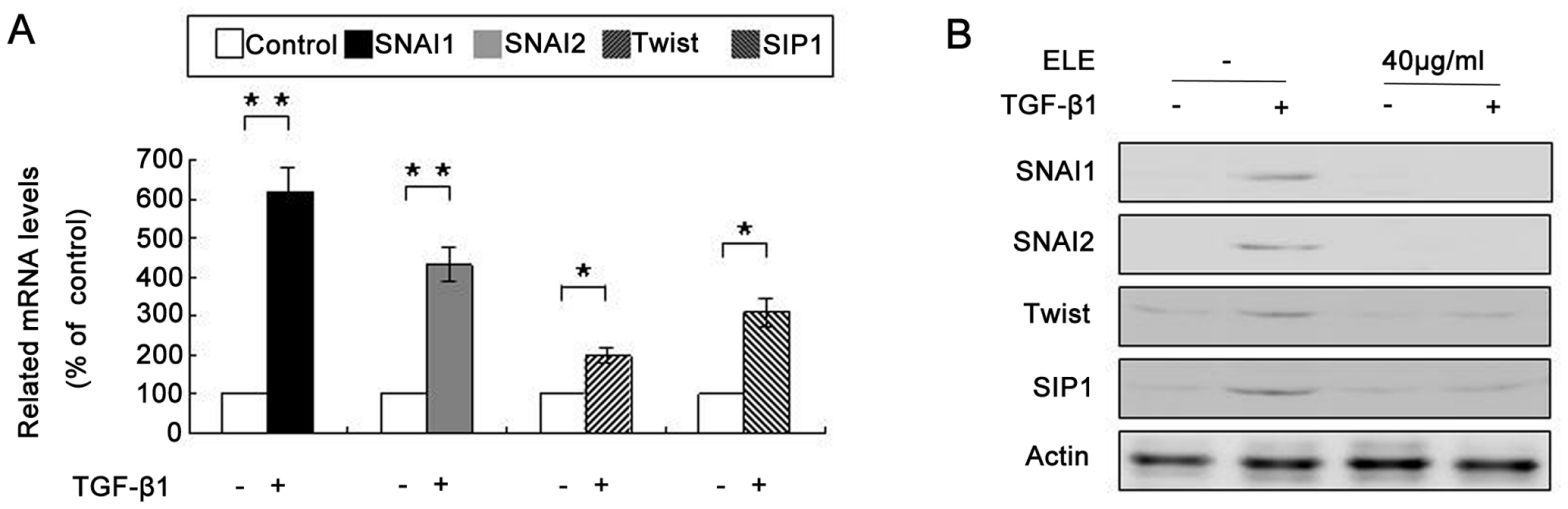
TGF-β1-mediated upregulation of nuclear transcriptional factors expression was blocked by ELE. (A) RT-PCR analyses showed that mRNA expression levels of transcriptional factors (SNAI1, SNAI2, TWIST and SIP1) were significantly increased in TGF-β1 treated MCF-7 cells (*: P<0.01, **: P<0.001). (B) Western immunoblot analysis showed that protein expression levels of SNAI1, SNAI2, TWIST and SIP1 were repressed by ELE treatment, especially in SNAI1 and SNAI2.

RT-PCR analyses showed that TGF-β1 treatment resulted in upregulation of SNAI1, SNAI2, TWIST and SIP1 mRNA expression in MCF-7 cells (Fig. 5A and Figure S2A). Consistent with the TGF-β1-dependent increase in mRNA expression of nuclear transcriptional factors, protein expression levels of these molecules were also elevated in response to TGF-β1 treatment. However, the increased expression of proteins was blocked using 40 µg/ml ELE (Fig. 5B).

### ELE Modulates TGF-β1-dependent Smad3 Expression and Phosphorylation

**Figure 6:**
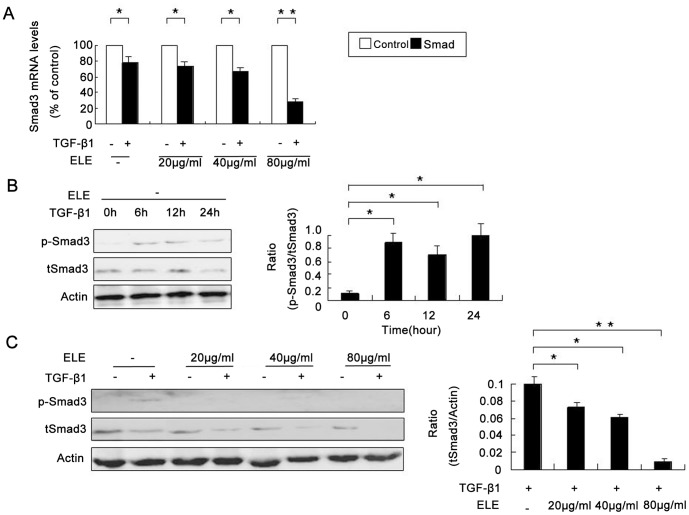
ELE facilitates TGF-β1-mediated downregulation of Smad3 expression but inhibits increased Smad3 phosphorylation. (A) RT-PCR analyses showed that TGF-β1 treatment led to downregulation of Smad3 mRNA expression, and ELE enhanced the reduction in Smad3 expression (*: P<0.01, **: P<0.001). (B) Western immunoblot analysis showed that the ratio of p-Smad3/total Smad3 was increased in a time-dependent manner with 10 ng/ml TGF-β1 (*:P<0.01), although expression levels of total Smad3 were slightly decreased. (C) TGF-β1-induced Smad3 phosphorylation was blocked by ELE and total Smad3 was further decreased in MCF-7 cells treated with TGF-β1 and ELE (*: P<0.01, **: P<0.001).

RT-PCR analyses showed that mRNA expression of Smad3 was inhibited using TGF-β1 in MCF-7 (P<0.01, Fig. 6A and Figure S2B). While 20 or 40 µg/ml ELE did not block TGF-β1-mediated downregulation of Smad3 expression, 80 µg/ml ELE significantly enhanced this reduction (P<0.001, Fig. 6A). As Smad3 is a downstream molecule in the TGF-β1 signalling pathway, the phosphorylation levels of Smad3 were significantly increased in response to TGF-β1 treatment (P<0.01, Fig. 6B), although total Smad3 protein levels were slightly decreased, consistent with the mRNA expression. Finally, we found that ELE significantly inhibited phophorylated Smad3 levels, and further reduced total Smad3 levels, at a concentration of 80 µg/ml (P<0.001, Fig. 6C).

## Discussion

TGF-β is a strong activator of EMT and involved in cancer progression and metastasis [36], [37], [38]. TGF-β is recognized as a master regulator of EMT in this phenotypic transition process [36]. The binding of TGF-β to its receptors leads to activation of a canonical Smad2/3-dependent pathway and subsequently phosphorylated Smad2/3 translocates to the nucleus where cell type-specific expression of TGF-β-responsive genes is regulated, including *SNAI1*, *SNAI2* and *SIP1* [39], [40]. In addition, there are non-canonical signalling pathways, including the MAP kinases ERK1/2, p38 MAPK, and JNK, the growth and survival kinases PI3K, AKT/PKB, and mTOR, and the small GTP-binding proteins Ras and RhoA [40], [41], [42].

In the TGF-β signalling pathway, there are several molecular targets with a potential for therapeutic interventions. One of the approaches is inhibition of TGF-β receptor I (TGFβRI) or TGF-β receptor II (TGFβRII) kinase activity, leading to a blockade of the phosphorylation of downstream effectors. However, there are a few clinical trials without success [43], [44]. In addition, a number of transcription factors that induce EMT through transcriptional repression of E-cadherin are also potential targets. While there is crosstalk among Snail family members and other transcription factors, including TWIST [45], ZEB1 [46] and SIP1 [47], knocking down Snail gene expression using RNA interference technology can effectively block cancer metastasis [48].

Since ELE was previously shown to regulate the expression and phosphorylation of kinase molecules, we hypothesized that ELE might affect EMT in breast cancer cells by modulating signalling proteins. To test this hypothesis, we initially established an EMT model using an epithelial breast cancer cell line, MCF-7, and examined the effect of ELE on the TGF-β1-dependent signalling pathway and the nuclear transcription factors SNAI1, SNAI2, TWIST and SIP1.

In agreement with previous studies that TGF-β1 induces morphological changes in epithelial cells, we found that MCF-7 treated with TGF-β1 became fibroblast-like similar to EMT, characterized by the expression of the epithelial markers (E-cadherin and β-catenin) and the mesenchymal markers (N-cadherin and vimentin). In addition, this mesenchymal phenotype was associated with more migration and invasiveness capabilities. Surprisingly, ELE blocked the phenotypic changes and abilities of migration and invasion of TGF-β1-treated MCF-7 cells. This blockade may be partially mediated by ELE-dependent regulation of nuclear transcription factor expression and TGF-β1-induced Smad3 expression and phophorylation.

The results of the invasion assay showed a slight increase in MCF-7 cells invading the Matrigel matrix when treated with ELE (compared to Figures 2B to 4B). We have previously reported that ELE treatment led to upregulated expression of the oestrogen receptor alpha (ERalpha) though repressing activity of the Ras/MARK/ERK pathway in breast cells [49]. As ERalpha is implicated in tumor invasion through the regulation of miRNA families, it is possible that upregulation of ERalpha expression may facilitate expression of its target miRNAs that are involved in cell invasion [50]. Since estrogen opposes TGF-β1 effects in MCF-7 cells, it is likely that ELE-induced expression of ERalpha may antagonize the TGF-β1-mediated EMT [51]. However, a more detailed mechanism by which ELE alone causes a small fraction of cells to invade requires further investigation.

Finally, our data provide a potential mechanism whereby ELE blocks TGF-β1-induced EMT in breast cancer cells *in vitro*. ELE-dependent inhibition of TGF-β1 effect is mediated by a reduction in Smad3 phosphorylation, enhanced downregulation of Smad3 expression and repressing expression of nuclear transcription factors.

## Supporting Information

Figure S1 **ELE MTT assay.** MCF-7 cells were treated with the indicated concentrations of ELE with or without 10 ng/ml TGF-β1. The IC50 for ELE was 534 µg/ml in TGF-β1-treated MCF-7 cells and 275 µg/ml in control cells, respectively.
(TIF)

Figure S2 **RT-PCR analyses of mRNA expression levels of nuclear transcriptional factors and Smad3.** RT-PCR analyses of mRNA expression levels of nuclear transcriptional factors (A) and Smad3 (B) in MCF-7 cells that were treated with or without 10 ng/ml TGF-β1 in the indicated concentrations of ELE.
(TIF)
