# Supplementary figures and images for: Beta-Elemene Blocks Epithelial-Mesenchymal Transition in Human Breast Cancer Cell Line MCF-7 through Smad3-Mediated Down-Regulation of Nuclear Transcription Factors
Source: PLoS One. 2013 Mar 14;8(3):e58719. doi: 10.1371/journal.pone.0058719 (PMC3597725; doi:10.1371/journal.pone.0058719)

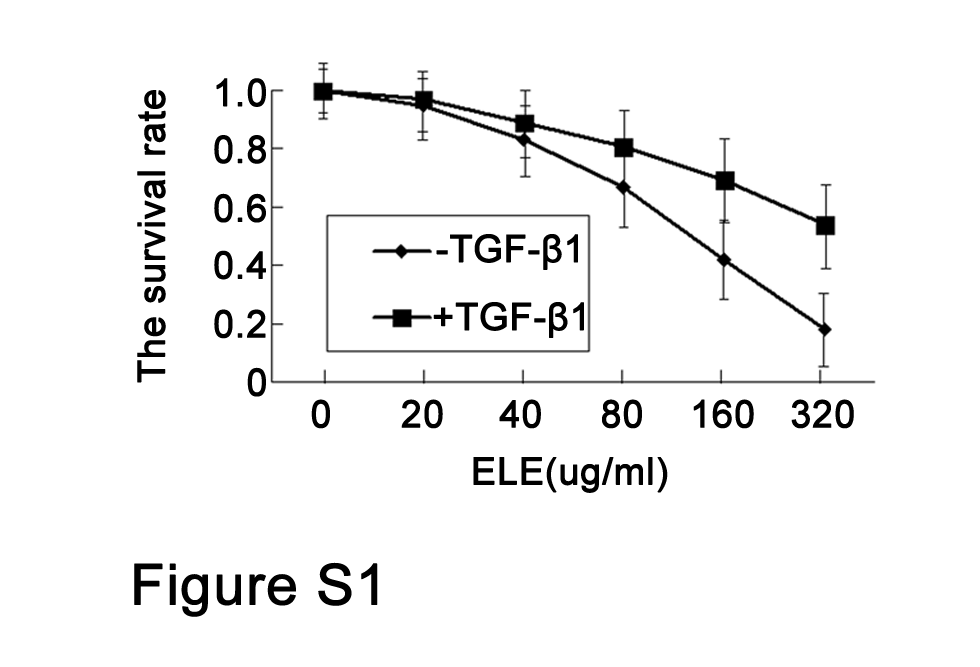

Supplement: Figure S1 — ELE MTT assay. MCF-7 cells were treated with the indicated concentrations of ELE with or without 10 ng/ml TGF-β1. The IC50 for ELE was 534 µg/ml in TGF-β1-treated MCF-7 cells and 275 µg/ml in control cells, respectively. (TIF) [file pone.0058719.s001.tif]

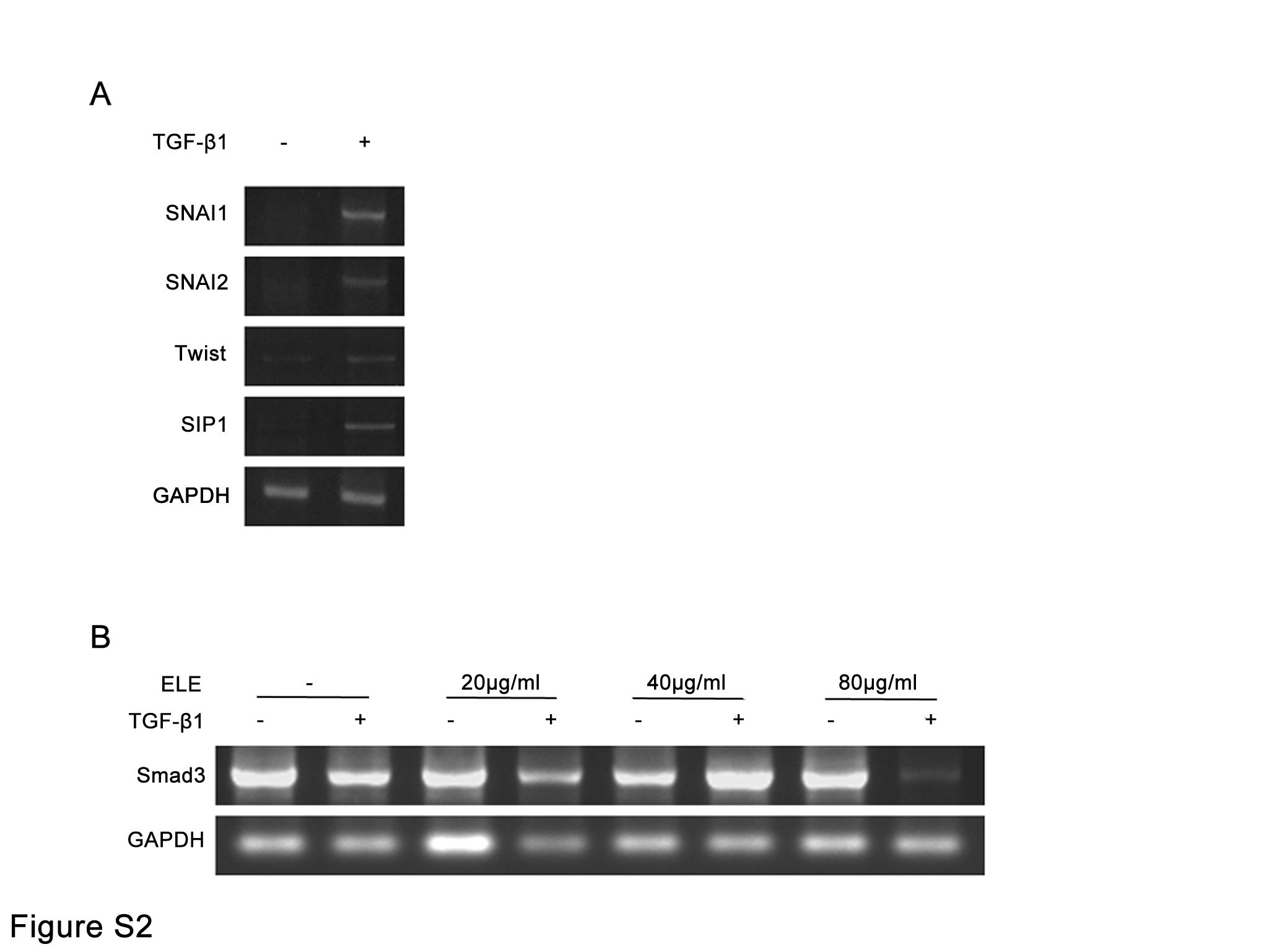

Supplement: Figure S2 — RT-PCR analyses of mRNA expression levels of nuclear transcriptional factors and Smad3. RT-PCR analyses of mRNA expression levels of nuclear transcriptional factors (A) and Smad3 (B) in MCF-7 cells that were treated with or without 10 ng/ml TGF-β1 in the indicated concentrations of ELE. (TIF) [file pone.0058719.s002.tif]
